# Factors associated with hospitalization in bronchiectasis exacerbations: a one-year follow-up study

**DOI:** 10.1186/s12931-017-0659-x

**Published:** 2017-09-30

**Authors:** Rosario Menéndez, Raúl Méndez, Eva Polverino, Edmundo Rosales-Mayor, Isabel Amara-Elori, Soledad Reyes, Tomás Posadas, Laia Fernández-Barat, Antoni Torres

**Affiliations:** 1 Pneumology Department, Hospital Universitario y Politécnico La Fe / Instituto de Investigación Sanitaria (IIS) La Fe. University of Valencia, Valencia, Spain; 2 Institut D’ Investigacions Biomèdiques August Pi I Sunyer (IDIBAPS), Barcelona, Spain; 3 Pneumology Department, Hospital Clínic / Institut D’ Investigacions Biomèdiques August Pi I Sunyer (IDIBAPS). University of Barcelona, Barcelona, Spain; 4 Centro de Investigación Biomédica En Red-Enfermedades Respiratorias (CIBERES, CB06/06/0028), Madrid, Spain

## Abstract

**Background:**

Bronchiectasis (BE) is a chronic structural lung disease with frequent exacerbations, some of which require hospital admission though no clear associated factors have been identified. We aimed to evaluate factors associated with hospitalization due to exacerbations during a 1-year follow-up period.

**Methods:**

A prospective observational study was performed in patients recruited from specialized BE clinics. We considered all exacerbations diagnosed and treated with antibiotics during a follow-up period of 1 year. The protocol recorded baseline variables, usual treatments, Bronchiectasis Severity Index (BSI) and FACED scores, comorbid conditions and prior hospitalizations.

**Results:**

Two hundred and 65 patients were recruited, of whom 162 required hospital admission during the follow-up period. Independent risk factors for hospital admission were age, previous hospitalization due to BE, use of proton pump inhibitors, heart failure, FACED and BSI, whereas pneumococcal vaccination was a protective factor. The area under the receiver operator characteristic curve (AUC) was 0.799 for BSI model was 0.799, and 0.813 for FACED model.

**Conclusions:**

Previous hospitalization, use of proton pump inhibitors, heart failure along with BSI or FACED scores is associated factors for developing exacerbations that require hospitalization. Pneumococcal vaccination was protective. This information may be useful for the design of preventive strategies and more intensive follow-up plans.

## Background

Bronchiectasis (BE) is a chronic structural respiratory disease characterized by dilated bronchi that courses with exacerbations that may require hospital admission [1, 2]. Although the incidence of BE is not well known, the average annual age-adjusted hospitalization rate was reported to be around 9.4 hospitalizations per 100,000 population in Germany, [3] and 16.5 in the United States [4]. Hospitalizations were higher among women and in the >60 year age group, though no clear predictors of hospital needs were identified. The average rate of exacerbations per year varies widely among patients and the causes remain unknown.

Exacerbations may lead to deterioration of lung function, [5] poor prognosis [6] and increased mortality [4, 7] and costs, [8] as in patients with other chronic respiratory diseases [9, 10]. In general, patients with advanced phases of disease and high Bronchiectasis Severity Index (BSI) or FACED scores have an average of two or more exacerbations per year [11], and the trend towards longer hospital stays [4, 12].

Few data are available on risk factors and patient characteristics in BE that might provoke exacerbations requiring hospital admission [13] apart from severity scales. This information may be useful for promoting strategies to prevent hospitalization and for personalized patient monitoring and management. Exacerbations requiring hospitalization are important endpoints for studies, as is their potential influence on worse quality of life [14] and early and long-term outcome [6]. In the EMBARC registry of BE patients, around one third of them require at least one hospitalization per year [15]. We hypothesized that several factors related to host characteristics, to comorbidities, to prior exacerbations, usual treatments along with BE scales must be associated with developing exacerbations requiring hospital admission.

The aim of our study was to evaluate factors associated with exacerbations requiring hospital admission, with regard to host characteristics, usual treatments, severity scores (FACED and BSI) and history of prior exacerbations, during a one-year follow-up period.

## Methods

### Study protocol

We conducted a prospective, observational study of adult bronchiectasis patients attended at the specialized outpatient clinics of two tertiary care university hospitals between 2011 and 2015 belonging to the Spanish National Health Service. Inclusion criteria included a compatible clinical history consistent of chronic sputum production and/or frequent respiratory infections with confirmed findings of bronchiectasis by computerized tomography (CT) scan of lungs performed prior to study recruitment. The investigation of the etiology of bronchiectasis was performed using a protocol in accordance to Spanish guidelines [16]. Exclusion criteria were: a) severe immunosuppression, as in solid-organ or bone-marrow transplantation or Human immunodeficiency virus infection/acquired immune deficiency syndrome (HIV/AIDS), or receiving chemotherapy or other immunosuppressive drugs (≥20 mg prednisone-equivalent per day for 2 weeks or more); b) active tuberculosis; c) cystic fibrosis (CF); and d) pulmonary interstitial disease. Patients signed the informed consent form (Biomedical research ethics committee Hospital La Fe 2011/0342), and after enrolment they were followed up for 1 year.

Data collected were demographic data, diagnosis of BE, comorbidities, smoking, alcohol intake, and vaccine status (flu and pneumococcal vaccines). Comorbid conditions recorded were diabetes, chronic obstructive pulmonary disease (COPD), asthma, chronic heart failure, myocardial infarction, prior tuberculosis, and renal, liver and cerebrovascular diseases. We recorded COPD as comorbidity similar to other studies [17] and we defined bronchiectasis associated with COPD in the presence of a smoking history of at least 10 pack-years with airflow obstruction (FEV_1_/FVC ratio < 0.7) according to the Global Initiative for Chronic Obstructive Lung Disease recommendations [18]. The association between BE and COPD is currently under an ongoing debate regarding the difficulties in its clarification [19–21].

Data related to previous chronic infections (defined according to Spanish guidelines), [16] number of exacerbations in the previous year, and bronchiectasis severity scores (BSI, FACED) [6, 22] were also recorded for all patients*.* Usual chronic and concomitant medications included bronchodilators, corticosteroids, theophylline, inhaled/nebulized antibiotics, proton pump inhibitors, long-term oxygen therapy, and mucolytic drugs in the last 6 months frame.

The microbiological diagnosis of exacerbation was performed with the following tests: sputum culture, urine (Binax Now for *S. pneumoniae* and *L. pneumophila* urinary antigen test), two blood samples, and nasopharyngeal swabs (for influenza A and B, parainfluenzae, syncytial respiratory virus, and adenovirus). Sputum and bronchoalveolar lavage (BAL) were processed for Gram and Ziehl–Neelsen strains and for cultures of bacterial, fungal and mycobacterial pathogens. Sputum samples were considered acceptable if there were more than 25 leukocytes and fewer than 10 squamous cells per low-power microscope field. Invasive samples, as BAL, were obtained only if requested by the attending physician. In outpatients exacerbations microbiological tests included sputum culture and any other additional test according to physician decision. The microbiological etiology of exacerbation was defined as any positive result from the microbiological investigation, as per previous publications [23].

### Exacerbation definition and follow-up

In accordance with Spanish guidelines [16] exacerbations were defined as follows: acute change in sputum characteristics (increased volume, change of viscosity, purulence) with or without increased dyspnea after ruling out any other causes, along with the requirement of a new antibiotic treatment prescribed in our specialized clinic and/or unscheduled admission to hospital. Exacerbations presenting with a new infiltrate in chest-X-ray were also recorded. The decision of hospital admission was made by the attending physician at emergency department without pre-prepared criteria and considering acute findings -clinical, analytical and radiographic- consistent with severe exacerbation [16].

Inpatients were followed up in visits to the specialized clinic at 30 days, 90 days and 1 year after discharge. For outpatients, follow-up was performed on day 7, 30 days, 90 days and 1 year. A telephone interview was conducted for patients who did not go to the visits to assess follow-up outcomes.

### Statistical analysis

#### Univariable analysis

Statistical analysis was performed using the SPSS 20.0 software program (IBM Corporation, Armonk, NY, USA). Qualitative variables were compared using the χ^2^ test. Quantitative variables were analyzed using the ANOVA test or the Kruskal-Wallis test. Values of p ≤0.05 were considered statistically significant. The total cohort of patients was separated in two groups: the study group comprised patients requiring one or more hospital admission due to exacerbation during the one-year follow-up period, and the control group those that did not require hospitalization.

### Multivariable analysis

Two logistic regression analyses were performed to predict hospital admission during 1-year follow-up as the dependent variable (≥1 admissions during 1 year follow-up vs. no hospital admission). Independent variables were the ones with *p* < 0.1 in the univariable analysis and those considered clinically relevant such as comorbid conditions and usual treatments. To address colinearity among variables highly correlated with each other, we used the variation inflation factor. Since both BSI and FACED are prognostic BE scales highly correlated they were introduce in the multivariate analysis separately; BSI in the first model and FACED in the second. The Hosmer and Lemeshow goodness-of-fit test was used to evaluate the adequacy of the models [24]. The area under the receiver-operator characteristic curve (AUC) for the models was also calculated.

## Results

### Patient characteristics

**Fig. 1:**
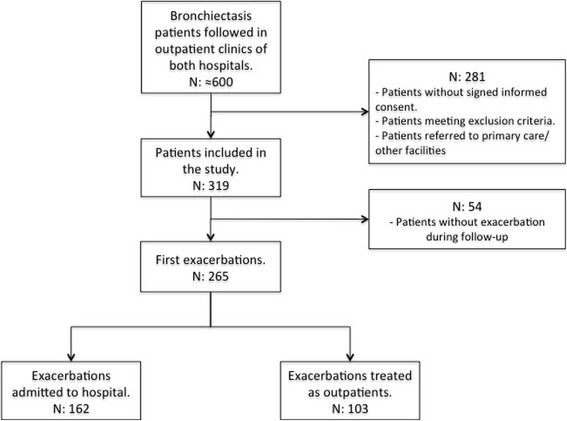
Flowchart

The cohort consisted of 319 patients followed up for 1 year and separated in two subsets: patients treated as outpatients and those admitted to hospital at least once during the follow-up period (Fig. 1). The mean age for the whole cohort was 68.4 (65 years in outpatients vs 73 in inpatients, *p* < 0.001); 106 (40%) were male and 159 were female. The distribution according to the FACED scale was 133 (50.2%) mild, 89 (33.6%) moderate, 43 (16.2%) severe, and according to the BSI scale 47 (17.7%) mild 73 (27.5%) moderate, 145 (54.7%) severe.

Three patients died during the exacerbation and 12 more in the 1-year follow up period; mortality was higher in hospitalized patients (p: 0.04).

### Risk factors for hospital admission. Clinical features of hospitalized exacerbation

#### Univariable analysis

**Fig. 2:**
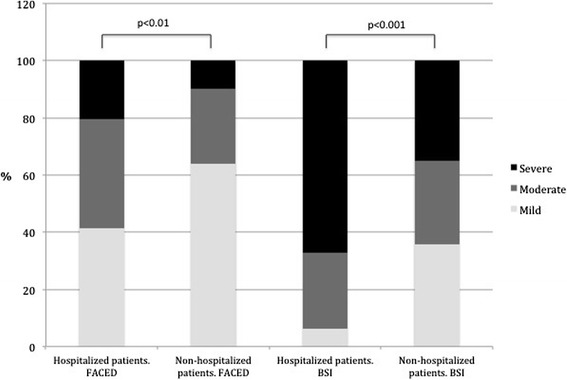
Distribution of subset of patients in the cohort (≥1 hospital admission vs no admissions) according to FACED and BSI scores

A total of 162 patients were admitted to hospital during the follow-up period. Forty five patients had respiratory failure, 133 worsening dyspnea, 60 new findings in chest radiograph, 53 tachypnea ≥22, 57 tachycardia ≥100 and 51 fever ≥38 °C. Their characteristics, etiological BE diagnosis, previous bronchial chronic infection, number of exacerbations, number of prior antibiotic treatments, usual concomitant medications and severity scores are described in Table 1. Hospitalized patients were older, with more chronic comorbid conditions but with similar colonization rates. The median days from onset of exacerbation to first visit was 4 days (IQR:2–10) in outpatients (data obtained in 49 out of 70) and 3 (IQR 2–7) in those hospitalized (data from 139 out of 162 patients), p:0.1. Prognostic scales according to hospital admission are shown in Fig. 2. The distribution of the etiology of BE is shown in Table 2.

**Table 1:** Patient Characteristics, Comorbid Conditions, Usual Treatments, Prior Colonization Status and Scales According to Hospitalization

Characteristics	Hospitalization at one year follow-up		
	No	Yes	*p*-value
Total No.	103 (38.9)	162 (61.1)	
Demographic data			
Age (years)	65 (55–73)	73 (68–80)	<0.001
Gender			
Male	27 (26.2)	79 (48.8)	<0.001
Female	76 (73.8)	83(51.2)	
Smoker or former smoker	48 (46.6)	77 (47.5)	0.883
Alcohol abuse	4 (3.9)	7 (4.3)	0.862
Flu vaccine	75 (72.8)	109 (67.3)	0.341
Pneumococcal vaccine	59 (57.3)	64 (39.5)	0.005
PPSV23	48 (46.6)	54 (33.3)	0.030
PCV13	11 (10.7)	10 (6.2)	0.186
Comorbid condition			
Diabetes mellitus	7 (6.8)	34 (21)	0.002
Myocardial infarction	2 (1.9)	16 (9.9)	0.012
Heart failure	3 (2.9)	31 (19.1)	<0.001
Dementia	3 (2.9)	6 (3.7)	0.729
COPD	11 (10.7)	54 (33.3)	<0.001
Asthma	11 (10.7)	15 (9.3)	0.705
Renal disease	4 (3.9)	10 (6.2)	0.417
Liver disease	3 (2.9)	13 (8)	0.089
Radiology			
Cystic bronchiectasis	6 (5.8)	11 (6.8)	0.755
Colonization			
*Pseudomonas aeruginosa* colonization	34 (33)	66 (40.7)	0.206
Colonization by other microorganism	23 (22.3)	28 (17.3)	0.310
Colonization by MDR microorganism	7 (6.8)	23 (14.2)	0.064
Treatment			
Long-acting β-agonist	79 (76.7)	125 (77.2)	0.931
Long-acting anticholinergic	40 (38.8)	100 (61.7)	<0.001
Inhaled corticosteroids	75 (72.8)	123 (75.9)	0.570
Long term oral antibiotics	11 (10.7)	16 (9.9)	0.833
Inhaled/Nebulized antibiotic	17 (16.5)	29 (17.9)	0.770
Mucolytics	28 (27.2)	51 (31.5)	0.456
Proton pump inhibitor	26 (25.2)	100 (61.7)	<0.001
Chronic oxygen therapy	4 (3.9)	25 (15.4)	0.013
Statins	21 (20.4)	26 (16)	0.367
Regular chest physiotherapy	33 (32)	42 (25.9)	0.282
Immunosuppressors	1 (1)	4 (2.5)	0.382
NIMV	0 (0)	4 (2.5)	0.108
History of exacerbations			
Hospitalization last year due to BE	23 (22.3)	97 (59.9)	<0.001
Previous hospitalization due to BE at anytime	33 (32)	98 (60.5)	<0.001
Previous history of pneumonia	39 (37.9)	85 (52.5)	0.020
Exacerbation last year	74 (71.8)	118 (72.8)	0.860
No. exacerbations last year	1 (0–2)	1 (0–2)	0.482
No. antibiotic treatments last year >2	19 (18.4)	39 (24.1)	0.280

Data are presented as n (%) or median (interquartile range)

Alcohol abuse: More than 80 g/day

*PPSV23* pneumococcal polysaccharide vaccine

*PCV13* pneumococcal conjugate vaccine

*COPD* chronic obstructive pulmonary disease

*MDR* multidrug-resistant

*NIMV* non-invasive mechanical ventilation

*P*-value: The χ2 test was performed for categorical data and the Mann-Whitney U test was performed for continuous data

**Table 2:** Etiology of bronchiectasis and 1 year follow-up hospitalization

Etiology of bronchiectasis			
	1 year follow-up hospitalization		*p*-value
	No	Yes	
Idiopathic	44 (42.7)	48 (29.6)	0.005
Post-infectious^a^	30 (29.1)	51 (31.5)	
COPD^b^	10 (9.7)	42 (25.9)	
Others	19 (18.4)	21 (13)	

Data are presented as n (%)

*COPD* Chronic obstructive pulmonary disease

P-value: the Mann-Whitney U test was performed for continuous data

^a^A diagnosis of postinfective bronchiectasis was made if the patient reported a history of symptoms due to bronchiectasis with an onset after a severe respiratory infection, such as pneumonia or tuberculosis, according to clinical judgment and regardless of the latency between the event and the occurrence of symptoms of bronchiectasis [38]

^b^Bronchiectasis associated with COPD was diagnosed in the presence of a smoking history of at least 10 pack-years with airflow obstruction (FEV1/FVC ratio < 0.7) according to the Global Initiative for Chronic Obstructive Lung Disease [20]

### Multivariable statistical analysis

Four independent risk factors and one protective factor were identified for predicting hospital admission at 1-year follow-up in the model adjusted by BSI score (Table 3) (FEV1, previous hospitalization due to BE and *P. aeruginosa* colonization were excluded by colinearity). Five risk factors and one protective factor were identified in the model adjusted by FACED score (FEV1 and *P. aeruginosa* colonization excluded). The AUC was 0.799 (95% CI: 0.745–0.853) for BSI model and 0.813 (0.760–0.866) for FACED model.

**Table 3:** Predictors of hospital admission: univariable and multivariable analysis

	Univariable analysis		Multivariable analysis			
	OR (95% C.I.)	*P*-value	BSI model OR (95% C.I.)	*P*-value	FACED model OR (95% C.I.)	*P*-value
Age	1.07 (1.04–1.08)	<0.001	1.01 (0.99–1.04)	0.270	1.03 (1.01–1.07)	0.021
Male	2.68 (1.57–4.58)	<0.001	1.27 (0.57–2.82)	0.559	0.16 (0.53–2.54)	0.702
Pneumococcal vaccine	0.49 (0.29–0.80)	0.005	0.37 (0.19–0.70)	0.003	0.40 (0.21–0.74)	0.004
Diabetes Mellitus	3.64 (1.55–8.57)	0.003	1.62 (0.60–4.76)	0.355	1.57 (0.59–4.55)	0.380
Myocardial infarction	5.53 (1.24–24.60)	0.025	0.78 (0.14–6.92)	0.794	0.72 (0.13–6.06)	0.727
Heart failure	7.89 (2.34–26.54)	0.001	6.31 (1.55–43.07)	0.023	5.47 (1.36–37.23)	0.035
COPD	4.18 (2.06–8.47)	<0.001	2.06 (0.79–5.63)	0.145	2.42 (0.93–6.59)	0.074
Previous MDR colonization	2.27 (0.94–5.50)	0.070	0.97 (0.32–3.10)	0.956	1.16 (0.40–3.61)	0.791
Long-acting anticholinergic	2.54 (1.53–4.22)	<0.001	1.48 (0.74–2.92)	0.262	1.69 (0.87–3.30)	0.120
Proton pump inhibitor	4.78 (2.77–8.24)	<0.001	2.64 (1.35–5.26)	0.005	2.85 (1.48–5.59)	0.002
Chronic oxygen therapy	4.52 (1.52–13.39)	0.007	1.82 (0.46–9.47)	0.427	2.73 (0.70–14.05)	0.179
Previous hospitalization due to BE at any time	3.25 (1.93–5.46)	<0.001	–	–	2.63 (1.36–5.17)	0.005
Previous history of pneumonia	1.81 (1.095–3.00)	0.021	1.55 (0.81–2.99)	0.185	1.41 (0.73–2.75)	0.307
FACED	5.22 (2.97–9.20)	<0.001	–	–	0.97 (0.78–1.22)	0.810
BSI	13.79 (6.53–29.13)	<0.001	1.20 (1.09–1.32)	<0.001	–	–

*BE* Bronchiectasis, *BSI* Bronchiectasis severity index, *FACED* F-FEV1, A-Age, C-*Pseudomonas aeruginosa* colonization, E-extension and D-Dyspnea, *C.I.* Confidence interval, *COPD* Chronic obstructive pulmonary disease, *MDR* Multidrug-resistant, *OR* Odds ratio

## Discussion

This study identified patient characteristics and clinical predictors of admission due to an exacerbation during a one-year-follow up period in BE patients. Age, heart failure, previous hospitalization due to BE, use of proton pumps inhibitors, and BE scales (FACED and BSI) were associated factors for hospital admission, whereas pneumococcal vaccination was a protective factor.

Patients with BE frequently present chronic infections by pathogens with exacerbations that may require hospital admission, although the associated factors that cause them are not clearly identified. The publication for defining BE exacerbation has just been published although it has not include criteria for hospitalization [25]. It has been suggested that these criteria may be similar to those of COPD [26]. Exacerbations may be caused by pneumonic or non-pneumonic episodes that are very difficult to distinguish in clinical practice without performing a CT-scan; this is why, in our study, we did not separate exacerbations on the basis of the appearance of new infiltrates. In a prior study, Polverino et al. [23] reported that BE was present in 3% of hospitalized community-acquired pneumonia (CAP) patients and that on average BE patients had two or three exacerbations per year. Our study found that patients requiring hospitalization were older, had more chronic diseases, more regular concomitant treatments and higher chronic *Pseudomonas* colonization.

We found five independent risk factors for exacerbations requiring admission to hospital: age, use of proton-pump inhibitors, previous BE hospitalization, heart failure and BE scales. An important factor such as chronic *Pseudomonas colonization* highly associated with requirement of hospitalization was not evaluated in the multivariable analysis due to the fact that it is included in the severity scales presenting high colineality [11]. Use of proton-pump inhibitors is recognized as a risk factor for the appearance of CAP, with a 1.5-fold increase, [27] and for COPD exacerbation [28]. Although the pathophysiological mechanisms have not been clearly defined, it has been suggested that CAP may develop due to the influence of proton-pump inhibitor (PPIs) on the gut microbiome [29]. In fact, PPIs modify the composition of the microbiome, reducing microbial abundance in gut and increasing levels of oral and upper gastro-intestinal tract commensals due to changes in pH. This alteration of microbiome could contribute to develop more severe exacerbations requiring hospital admission. The con formation of this hypothesis will require further investigations. Schuijt et al. [30] in a mouse model found that after the depletion of the gut microbiota, there was an increase in bacterial dissemination, inflammation and even organ damage. Purcell et al. [31] reported that in some patients acute BE exacerbations, their frequency, and episodes of clinical stability are correlated with a significantly different bacterial community structure, which is associated with the presence of particular taxa in non-cystic fibrosis bronchiectasis.

Airway reflux is quite prevalent in BE patients [32] and it is also a recognized risk factor for exacerbation, [33] just as it is in hospitalized patients with moderate-to-severe COPD [28]. In fact, symptoms of airway reflux independently predict severity and exacerbation frequency in BE [33]. However, our findings and those of prior publications are not sufficient to fully evaluate the impact of the disease and/or the use of PPIs; the role of airway reflux and its treatment with PPIs needs to be better clarified in BE patients in larger cohorts .

Previous hospitalization was an independent risk factor for a BE exacerbation requiring hospital admission. Prior hospitalization has been clearly identified as the most decisive risk factor for severe exacerbation in other chronic respiratory diseases [34]. Moreover, having been hospitalized for an exacerbation significantly increased the risk of mortality. Greater bronchial and systemic inflammation has been observed during exacerbations, [35] which probably contributes to perpetuating the infection-inflammation cycle and has a negative effect on prognosis. The percentage of females treated ambulatory was higher than males while in patients requiring hospital there were no gender differences. Male gender was associated with more severe disease, more comorbidities and higher *Pseudomonas* colonization similar to other studies [36, 37]. When adjusting by these factors, gender disappear as independent risk factor for admission. Ringshausen et al. [3]have also reported an increased in hospitalization among elderly men.

As might be expected, BE patients with higher BSI and FACED scores required more hospital admissions, regardless its recognized differences in classifying BE patients in the severe phases: -BSI classified more patients as severe than FACED- [38, 39] as confirmed in our study. The mathematical models retained identical risk and protective factors with minor changes in the OR when both scales (BSI or FACED) are evaluated separately. Recently the new E-FACED [40] and Exa-FACED scores [39], which incorporates the number of exacerbations, has demonstrated a better prognostic capacity for subsequent exacerbations and 1 year hospitalization. Recently, McDonnell et al. defined the Bronchiectasis Aetiology Comorbidity Index (BACI) in an elegant study [17] based on multimorbidity and its independent influence for mortality prediction. This score improves predictive capacity of other scales including the prediction for admission. Nevertheless, this score does not include data as vaccination and/or concomitant treatments, determinant predictors of hospitalization in other infectious lung diseases as CAP [41]. In our study, designed prior to publication BACI scale, we have found the independent effect of heart failure that is a frequent comorbid condition in patients with chronic respiratory diseases.

Vaccination against influenza and pneumococci are associated with better survival in patients with BE [42]. This protective factor was confirmed in exacerbations requiring hospital admission; to our knowledge, this has not been previously reported in BE patients. Pneumococcal vaccine has been recommended in the literature [43, 44] although specific studies for the BE population are lacking. Although, there are no data on the influence of vaccination on hospitalization in bronchiectasis, there is evidence of its influence on the occurrence of exacerbations [45].

Exacerbations admitted to hospital increased the risk of death during the study period compared with non-hospitalized exacerbations. Thereafter, an important aim should be to reduce the frequency and severity of exacerbations [46]. Severe exacerbations cause increased local and systemic inflammation, and repeat exacerbations probably contribute to a persistent cycle of inflammation and infection, with negative consequences for prognosis. In fact, after hospital admission, there were increases in both the percentage of new exacerbations at 30 days and mortality in the following year.

Our study has a number of limitations that should be mentioned. First, it is difficult to evaluate the presence of new infiltrates in BE exacerbations as pneumonia or merely exacerbation without performing a CT-scan. Our study was designed to follow our cohort in a “real life scenario”, that is, with chest X-rays instead of CT-scans, which are not feasible in a disease with frequent exacerbations. Our patients were recruited from specialized BE clinics representing a subset of patients with more advanced phases therefore our findings could not be generalizable for milder BE populations.

## Conclusion

In summary, our study identified a group of vulnerable BE patients likely to develop exacerbations requiring hospitalization during a one-year follow-up period adding some new valuable additional information to validated scales. These BE patients would probably benefit from a more extensive follow-up, high quality specialized care, intensified treatment, vaccine implementation and even novel therapies in order to improve prognosis. Identification of risk factors associated with hospital admission and more studies regarding the role of PPIs; may help to devise preventive strategies for improving the course of the disease and reducing both morbidity and the economic burden.

## Abbreviations

AUC: Area under the receiver-operator characteristic curve
BACI: Bronchiectasis aetiology comorbidity index
BAL: Bronchoalveolar lavage
BE: Bronchiectasis
BSI: Bronchiectasis severity index
CF: Cystic fibrosis
COPD: Chronic obstructive pulmonary disease
CT: Computerized tomography
FACED: F (forced expiratory volume in 1 s [FEV1]); A (age; C: chronic colonization by Pseudomonas aeruginosa [PA]); E (radiological extension [number of pulmonary lobes affected]); and D (dyspnea)
HIV/AIDS: Human immunodeficiency virus infection/acquired immune deficiency syndrome
PPIs: Proton-pump inhibitors
